# Highly Insulated Wall Systems with Exterior Insulation of Polyisocyanurate under Different Facer Materials: Material Characterization and Long-Term Hygrothermal Performance Assessment

**DOI:** 10.3390/ma13153373

**Published:** 2020-07-30

**Authors:** Emishaw Iffa, Fitsum Tariku, Wendy Ying Simpson

**Affiliations:** 1 Oak Ridge National Laboratory, Building Envelope & Urban Systems Research, Oak Ridge, TN 37831, USA; 2 British Columbia Institute of Technology, BCIT Building Science Centre of Excellence, Burnaby, BC V5G3H2, Canada; fitsum_tariku@bcit.ca (F.T.); wendy_simpson@bcit.ca (W.Y.S.)

**Keywords:** drying and wetting, hygrothermal performance, Polyisocyanurate board, moisture content, thermal performance, vapor permeability

## Abstract

The application of exterior insulation in both new construction and retrofits is a common practice to enhance the energy efficiency of buildings. In addition to increased thermal performance, the rigid insulation can serve to keep the sheathing board warm and serve as a water-resistive barrier to keep moisture-related problems due to condensation and wind-driven rain. Polyisocyanurate (PIR) rigid boards have a higher thermal resistance in comparison to other commonly used exterior insulation boards. However, because of its perceived lower permeance, its use as exterior insulation is not very common. In this study, the hygrothermal property of PIR boards with different facer types and thicknesses is characterized. The material data obtained through experimental test and extrapolation is used in a long term hygrothermal performance assessment of a wood frame wall with PIR boards as exterior insulation. Results show that PIR with no facer has the smallest accumulated moisture on the sheathing board in comparison to other insulation boards. Walls with a bigger thickness of exterior insulation perform better when no vapor barrier is used. The PIR exterior insulation supports the moisture control strategy well in colder climates in perfect wall scenarios, where there is no air leakage and moisture intrusion. In cases where there is trapped moisture, the sheathing board has a higher moisture content with PIR boards with both aluminum or fiberglass type facers. An innovative facer material development for PIR boards can help efforts targeting improved energy-efficient and durable wall systems.

This manuscript has been authored in part by UT-Battelle, LLC, under contract DE-AC05–00OR22725 with the US Department of Energy (DOE). The US government retains and the publisher, by accepting the article for publication, acknowledges that the US government retains a nonexclusive, paid-up, irrevocable, worldwide license to publish or reproduce the published form of this manuscript, or allow others to do so, for US government purposes. DOE will provide public access to these results of federally sponsored research in accordance with the DOE Public Access Plan (http://energy.gov/downloads/doe-public-access-plan).

## 1. Introduction

The ever-increasing demand from building codes for improved energy efficiency and society’s increasing awareness of environmental sustainability is driving the building construction and manufacturing industries to develop innovative solutions for durable, high-performance buildings. It is well documented that the application of exterior insulation increases the overall thermal performance of new construction and retrofits [1,2,3,4,5]. While studying the thermal performance of a building envelope, the moisture durability needs proper consideration as well [6,7] The moisture control performance requires additional investigation and researchers are examining the effects of rigid insulation on the durability of wall systems incorporating innovative materials and construction practices [8,9,10,11,12,13,14].

Among the commonly used insulation materials, Polyisocyanurate (PIR) is known for its higher R-value and consequently providing an increased energy efficiency when it is compared with most of the other foam-based insulation materials [15]. However, the building industry considers PIR as impermeable or semi-permeable, and its application as exterior insulation is limited. Regarding material characterization and hygrothermal performance assessment, relatively smaller research studies are reported in comparison to the thermal performance of exterior insulation. 

Burch and Desjarlais conducted a water vapor measurement test for PIR core and facer [16]. Their measurement shows that the glass-mat facers permeance varies from 600 ng/sm2.Pa (10 perm) to 2800 ng/sm2.Pa (49 perm). A study on advanced material preparation and characterization of PIR foams is an active research topic. Kosmela et al. have found that the addition of up to 30% by weight of bio-polyol, instead of foams prepared solely with a petrochemical polyol, have increased the reactivity of the polyol mixture in rigid Polyurethane-Polyisocyanurate(PUR-PIR) foams, which in turn has enhanced the thermal performance of the rigid foam [17]. Borowiciz et al. have also found that PIR foams modified with bio-polyol based on mustard seed oil have lowered the thermal conductivity and water absorption [18]. Berardi and Madzarevic analyzed the aging behavior and tested the blowing agent concentration of a PIR foam. A decrease in 11% and 85% of a blowing agent is measured from the aged PIR foams [19]. 

The closed cells created during PIR manufacturing are filled with the vaporized blowing agent during the foaming reaction [20]. To keep the blowing agent from migrating out and in return affecting the R-value of the PIR foam, different types of facers are used during PIR production [20,21,22,23]. Mackaveckas et al. reported on the influence of different PIR facings on thermal performance. Their findings show a significant heat loss in PIR insulation boards with aluminum facing at wall corners [24].

The main purpose of this study is to investigate the long-term hygrothermal performance of wall systems with PIR exterior insulation. To study the optimal use of the PIR insulation board, the thickness and type of facer materials were varied and their performance under different scenarios: varying moisture loads, vapor barrier applications, and different climates were investigated. Their performance was compared with another exterior rigid insulation board, extruded polystyrene (XPS).

The material characterization for the PIR took place at BCIT’s Building Science Centre of excellence (BSCE). The measured thermal and hygrothermal material properties include density, thermal conductivity, heat capacity, sorption isotherm, vapor permeability, water absorption coefficient, and porosity. The next sections of this paper discuss the experimental test, the simulation setup, results obtained, and the conclusions based on experiment and simulation results.

## 2. Hygrothermal Property Characterization of PIR Foam with Different Facers

To study the long-term performance of hygrothermal simulation performance of PIR as an exterior insulation under different climates, a hygrothermal material property characterization of three types of PIR products is conducted: fiberglass-faced PIR, PIR with aluminum facer and an unfaced PIR insulation. A series of laboratory measurements are carried out to characterize the hygrothermal properties of a rigid PIR insulation board with different facing materials. The hygrothermal properties measured are density, thermal conductivity, specific heat capacity, sorption isotherm, water vapor permeability, water absorption coefficient, and porosity. Measurements are conducted in accordance with the ASTM Standards [25,26,27,28]. The measurement procedures, standards used and material characterization results are shown in the section below.

The PIR hygrothermal property characterization under different facer materials for PIR of thickness 12.7 mm (0.5 in.), 25.4 mm (1 in.) and 38.1 mm (1.5 in.) are measured through a laboratory test. The obtained properties are extrapolated for other thickness sizes. The measured hygrothermal properties of PIR core insulation, PIR with fiberglass facer, and PIR with foil-faced PIR insulation are presented below.

The density is determined from physical dimensions and oven-dry mass measurements of six specimens for all three PIR samples, as shown in Table 1.

The thermal conductivity of the PIR core board and with foil and fiberglass facers is carried out according to ASTM Standard C518: “Standard Test Method for Steady-State Thermal Transmission Properties by Means of the Heat Flow Meter Apparatus “using four – 30 cm × 30 cm specimens (average thickness 24.86 mm). The measurements are done at a temperature difference of 20 °C (68 °F) and a mean temperature of 24 °C (75.2 °F). Table 2 shows the average thermal conductivity and resistance values of the four PIR board samples for all types of facers. In addition, the maximum and the minimum measured values are also presented in the table. 

The heat capacity of the PIR for the three samples is determined using a LaserComp Fox heat flow meter (TA instruments, New Castle, DE, USA) and WinTherm32 software (TA instruments, New Castle, DE, USA) for analysis and determination of transient heat transfer through the specimens. Based on four samples measurements, both volumetric heat capacity and specific heat capacity are measured for all three types of PIR with different facers. Table 3 shows the average volumetric and heat capacity values of PIR boards.

Sorption Isotherm: PIR boards with fiberglass facer and no facer are used under this study. Because of its low permeability, the aluminum-faced PIR board is not investigated for its hygrothermal property characterization. The equilibrium moisture contents at 50%, 70%, 80%, 90% and 95% relative humidity (RH) conditions and saturation moisture content in 100% RH are determined according to ASTM Standard C1498: “Standard Test Method for Hygroscopic Sorption Isotherms of Building Materials”. For each test point, three specimens with dimensions of 100 mm × 100 mm with a thickness of 24 mm are used. The measurements are carried out in controlled climatic chambers that are maintained at a constant temperature of 23 °C and the desired relative humidity set point. A water immersion test is used to determine the capillary saturation moisture contents of the samples. Table 4 shows the measured equilibrium moisture contents of PIR board specimens at different relative humidity.

The water vapor permeability of PIR insulation is determined according to ASTM Standard E-96: “Standard Test Methods for Water Vapor Transmission of Materials”. Using climatic chambers with wet cups and dry cups methods, the water vapor transmission rates of PIR samples are determined. The climatic chambers are set at 50%, 70% and 90% relative humidity and 23 °C temperature. The dry (0%) and wet (100%) relative humidity conditions in the test caps are provided by calcium chloride (CaCl_2_) desiccant and distilled water, respectively. For each test, three circular specimens of 11.94 cm in diameter are used as a replica. The average water vapor permeability values of PIR boards at different mean sample relative humidity are shown in Table 5.

The water absorption coefficient of the PIR samples is determined according to ASTM Standard C1794: “Standard Test Methods for Determination of the Water Absorption Coefficient by Partial Immersion.” Three test specimens, 100 mm × 100 mm with a thickness of 37 mm each, are used for the measurements. The lab conditions are 33.7 ± 0.2 °C temperature and 39.8 ± 3% relative humidity. Water was maintained at 20.6 ± 0.5 °C. The measured water absorption coefficients of the PIR are found to be 0.0004 kg/(m^2^·s^1/2^) and 0.0007 kg/(m^2^·s^1/2^) for fiberglass facer and no facer PIR boards, respectively.

The porosity of PIR samples are obtained by determining the full saturation weight of the samples following the water immersion test procedure. Based on these measurements of three replica specimens, the measured porosity values of no facer and fiberglass-faced of PIR boards are 31.37 ± 1.34% and 20.51 ± 0.39%, respectively.

**Figure 1:**
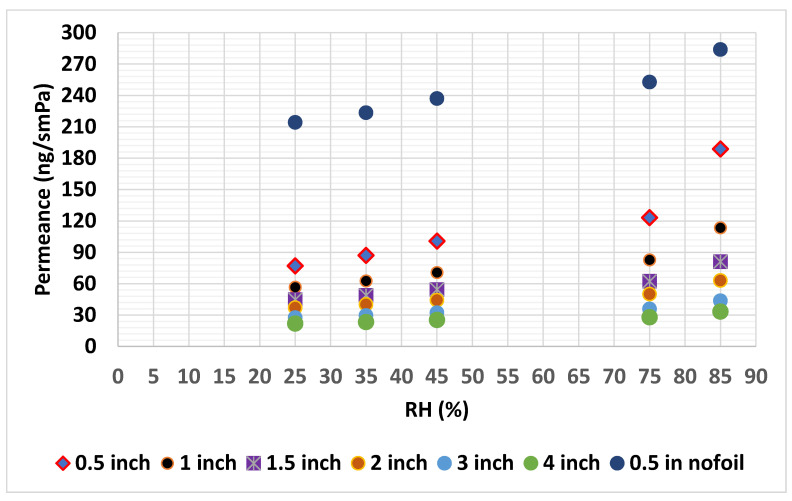
Water vapor permeance of fiberglass-faced Polyisocyanurate (PIR).

Since the hygrothermal property of the PIR with fiberglass is dependent on both the hygrothermal properties of PIR core and the facer material, a hygrothermal property of fiberglass-faced PIR and the PIR core is measured. By subtracting the water vapor resistance of the fiberglass-faced PIR from PIR core, the permeability of the fiberglass facer can be easily calculated. Once the independent permeability values of the facer material and PIR core are found, the overall permeability values of the fiberglass-faced PIR is computed. Figure 1 shows the water vapor transmission rate of fiberglass-faced PIR and PIR core at different thicknesses.

## 3. Hygrothermal Simulation

To study how the facing parts of rigid PIR exterior insulation affect the hygrothermal performance of a building envelope, two-year WUFI® simulations for a highly insulated rainscreen wall systems are conducted. This section discusses the simulation setup, initial and boundary conditions, simulation assumptions.

### 3.1. Simulation Set up

The indoor relative humidity and temperature conditions are set based on the ASHRAE standard 160 P Intermediate model. The input parameters varied to simulate the dynamic response of the wall system in different climates, with different types and thicknesses of exterior insulations, rain infiltration, and vapor barrier. The wall system that is considered for the study comprises the following layers of materials:

- regular Portland Stucco as an exterior cladding;
- 19 mm (¾ in.) rainscreen air gap;
- different types and thicknesses of exterior insulations;
- spun-bonded polyolefin as a sheathing membrane (as the second plane of protection from precipitation and water intrusion);
- plywood sheathing board;
- fiberglass insulation in 2 × 6 wood frame studs;
- 6 mm (1/4 in.) polyethylene sheet as a vapor and air barrier;
- gypsum board as an interior finishing layer

Table 6 shows the variation in simulation parameters during this modeling work. Three types of facing options (aluminum foil, fiberglass facer, and no facer) and two insulation thicknesses (2 in. and 4 in.) are considered in the study to evaluate the effect of PIR thickness and different facer materials on the overall hygrothermal performance of a wall system. Even though using a PIR product without facers is uncommon, incorporating this parameter shows how an innovative vapor-open facer product can enhance the hygrothermal performance of an envelope. While the material properties of the extruded polystyrene (XPS) were obtained from the WUFI^®^ database, the properties for the PIR insulation with different facer material properties are measured.

### 3.2. Boundary and Initial Conditions

The indoor conditions of relative humidity and temperature are set using the ASHRAE standard 160 P intermediate model. The external surface is exposed to the weather conditions of Vancouver, BC, to study the PIR performance with different facers and thickness under different moisture infiltration and combined effects of vapor barrier and exterior insulation. Weather data of two North American cities (Winnipeg, MB, Canada and Vancouver, BC, Canada) are used to study how a PIR exterior insulation of different facers performs from a hygrothermal perspective. The initial conditions of 20 °C and 80% RH are used for all wall component members and the simulation ran for two consecutive years. WUFI’s weather data for cities of Winnipeg, MB, and Vancouver is used and a cold year data is selected.

### 3.3. Modeling Assumptions

**Figure 2:**
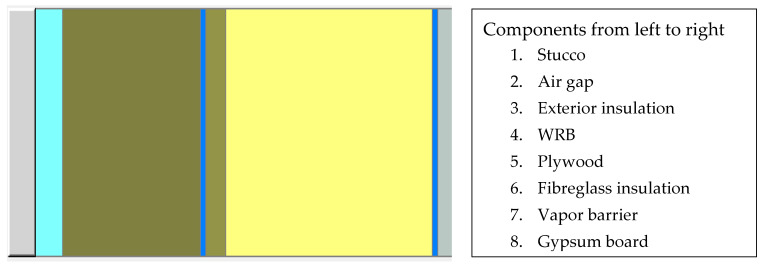
The wall assembly.

Figure 2 shows the wall assembly considered in the study. Continuity of vapor/air barrier (polyethylene sheet) is assumed to be maintained in the modeling. Therefore, there is no airflow through the wall system. The PIR boards with fiberglass and aluminum facers hygrothermal properties are lumped together based on the measured values of the core and facer material. The wall system was assumed to be with no deficiency and the layers of materials to be in perfect contact, exhibiting no dimensional physical change with time. The material properties of the wall components other than the PIR boards which are used in the simulation are shown in Table 7.

## 4. Hygrothermal Simulation Results and Discussions

In this section, based on the hygrothermal properties characterization of the PIR material, the long-term performance of a wood frame wall with PIR as exterior insulation under different thickness and facer materials for different climate zones is presented and discussed.

This section presents the results of a two-year hygrothermal assessment of four different types of exterior insulations, namely PIR with aluminum foil; PIR with fiberglass facer, PIR with no facer and XPS.

The effects of wind-driven rain, application of vapor barrier, different climates, and the overall performance of wall assemblies under different types and thicknesses of exterior insulations are presented.

### 4.1. Effect of Wind-Driven Rain

The Vancouver weather, a climate zone known for its heavy annual rainfall, is used to analyze how moisture infiltrated into an interior wall system due to wind-driven rain can affect the overall performance of a wood frame wall with exterior insulation.

**Figure 3:**
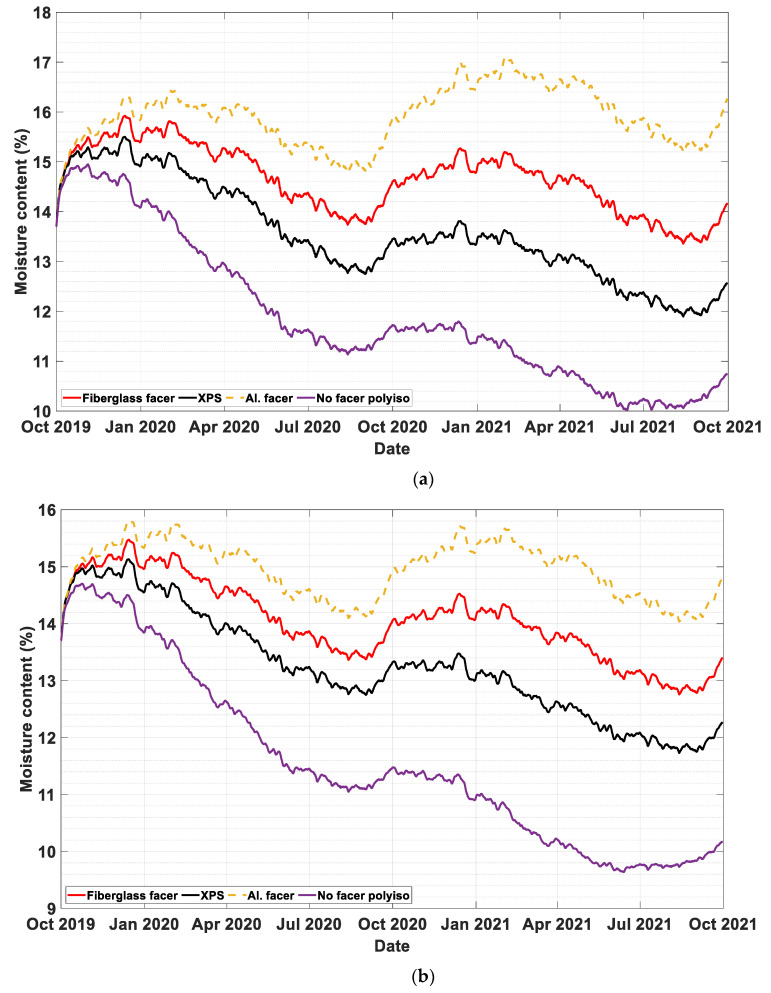
The moisture content of plywood with 0% rain infiltration in wall systems with (**a**) two-inch (**b**) four-inch exterior insulations.

**Figure 4:**
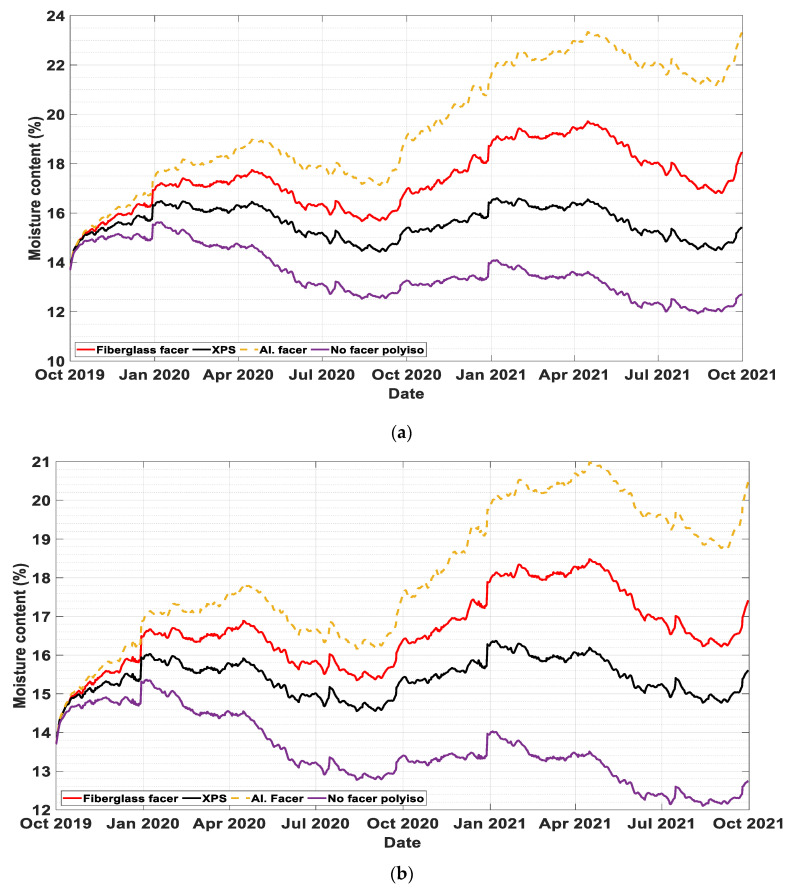
The moisture content of plywood with 0.1% rain infiltration in wall systems with (**a**) two-inch (**b**) four-inch exterior insulations.

**Figure 5:**
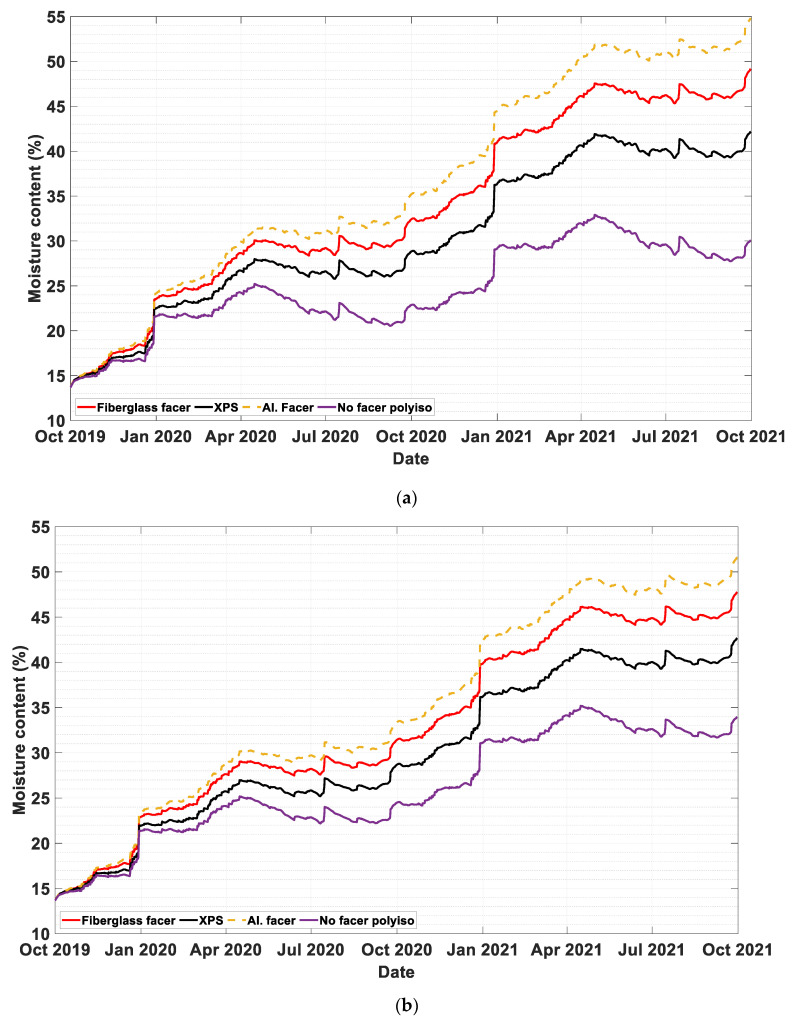
The moisture content of plywood with 0.5% rain infiltration in wall systems with (**a**) two-inch (**b**) four-inch exterior insulations.

Rain infiltration percentages of 0%, 0.1%, and 0.5% are assumed to reach the sheathing board (plywood). Figure 3a, Figure 4a and Figure 5a show the dynamic moisture content of plywood in wall systems with two-inch exterior insulation with different rain infiltration percentages. Similarly, Figure 3b, Figure 4b and Figure 5b represent moisture content values of plywood in wall systems with four-inch exterior insulation at 0%, 0.1%, and 0.5% infiltration percentages. In all cases, constant cavity ventilation of 100 ACH is assumed.

In most cases, the performance ranking from the least to the most accumulation of moisture on the plywood during the simulation period was a PIR with no facer, XPS, PIR with fiberglass facer, and PIR with aluminum foil. In cases without rain infiltration, starting from the last quarter of the second year, the wall with PIR exterior insulations without facer outperformed the other walls with different exterior insulation by a bigger margin.

In cases where the rain infiltration was not the dominant moisture source, for all wall systems and exterior insulation types, envelope systems with four-inch insulation performed better than envelope systems with two-inch insulation. This is because the thicker exterior insulation keeps the plywood warmer, minimizing the potential for condensation.

The moisture content of the plywood remained at an acceptable level for all wall systems when there is no rain infiltration. In simulation cases where 0.1% of the wind-driven rain was added in the plywood, all wall systems, except the wall with PIR with aluminum foil insulation, remained below the critical moisture level of 18%, for both 2 in and 4 in continuous simulation as shown in Figure 4a,b, respectively. This result shows if the moisture infiltration is managed to an acceptable limit most foam insulations with the exception of vapor impermeable foam insulation can be applied. 

To simulate an extreme case scenario, a rain infiltration of 0.5% is assumed. As Figure 5a,b show, most of the wall systems were subjected to very high moisture accumulation in the sheathing board. In this case the moisture control performance of the wall systems has failed in both 2-in and 4-in continuous insulations of the all simulated foam boards.

### 4.2. Combined Effects of Vapor Barrier and Exterior Insulation

Many building codes require an application of a vapor barrier to be used in building envelopes to enhance the moisture durability of wall systems. This study examines if the PIR exterior insulation can serve as a vapor barrier and what are the combined effects of vapor barrier and exterior insulation in wall systems.

**Figure 6:**
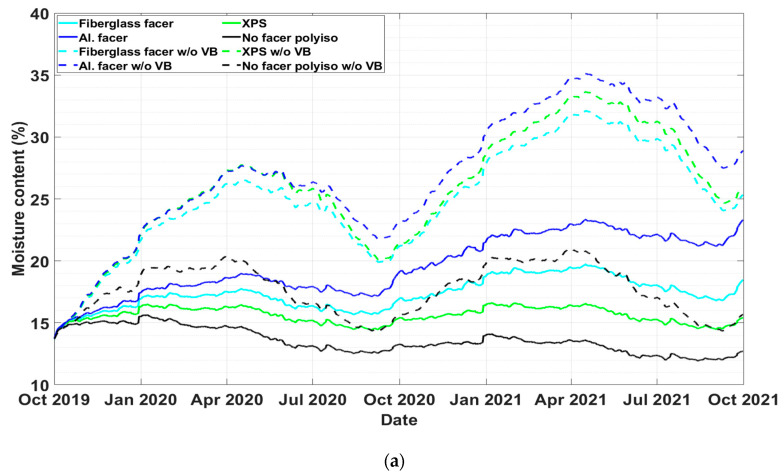

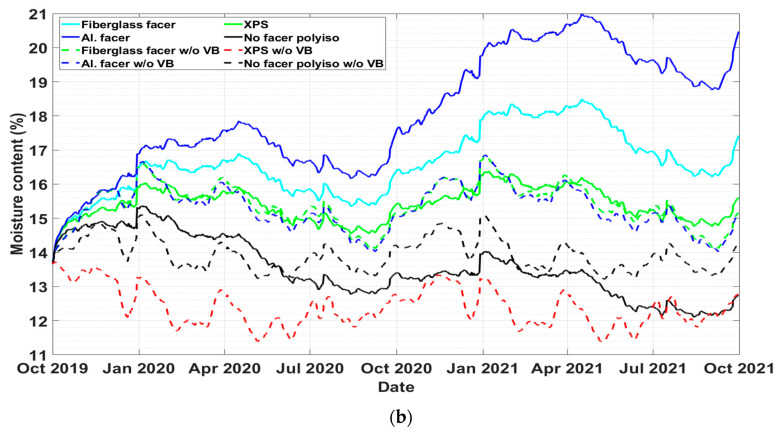
The moisture content of plywood in wall systems with and without vapor barrier and (**a**) two-inch and (**b**) four-inch exterior insulation thicknesses.

Figure 6a,b show the effect of the vapor barrier on the hygrothermal performance of a wall system under different exterior insulations. Figure 6a shows when the wall includes a two-inch thick exterior insulation, the wall systems with vapor barrier performed better in all four exterior insulation cases. The walls with no vapor barrier and either exterior insulation of PIR with aluminum foil, PIR with fiberglass facing, or XPS registered a high moisture content. From the wall systems with the vapor barrier, the only wall exposed to a beyond-critical moisture content was the wall with PIR with aluminum foil insulation.

Figure 6b shows the insulation thickness increased from two inches to four inches for all four types of exterior insulations. In all types of exterior insulations, the wall systems without vapor barrier perform better than walls with a vapor barrier. This due to the thick exterior insulation facilitating a warm condition for the plywood, discouraging condensation. The lack of a vapor barrier provides a route for moisture from rain infiltration to dry to the inside. The plywood moisture content stays below 18% in all wall systems without a vapor barrier. 

### 4.3. Hygrothermal Performance of Exterior Insulations under Different Climates

**Figure 7:**
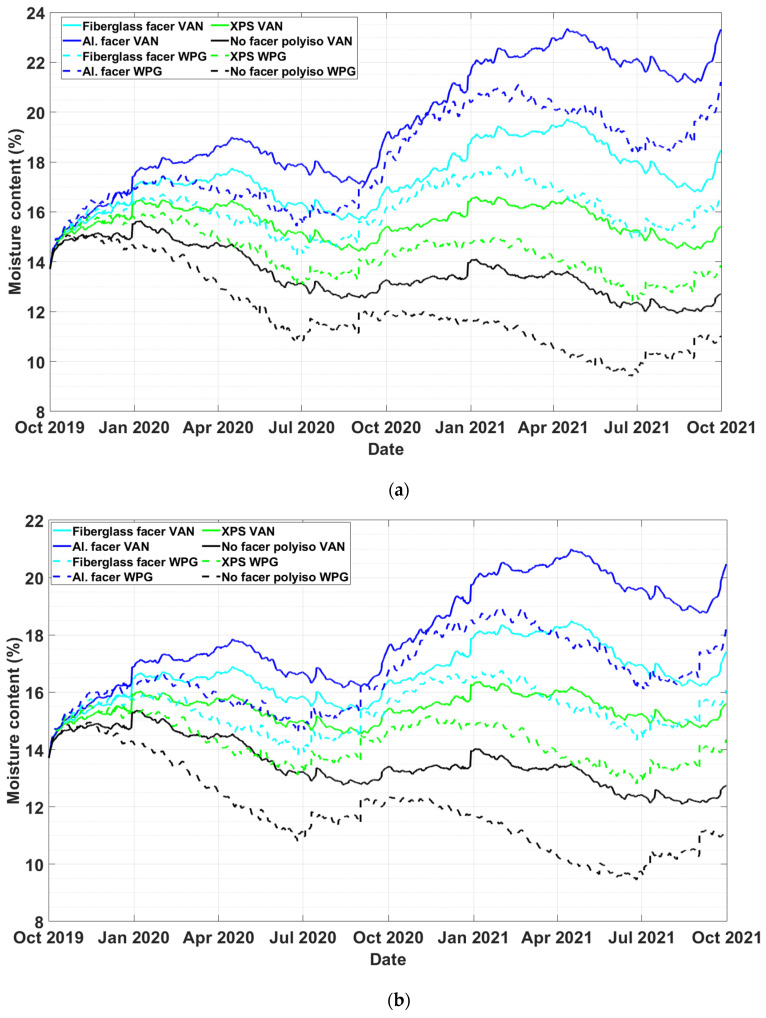
The moisture content of plywood in wall systems in Winnipeg, MB and Vancouver, BC with (**a**) two-inch and (**b**) four-inch exterior insulation thicknesses.

This study looked at the hygrothermal performance of the exterior insulation under the different climates of the cities of Winnipeg, MB and Vancouver, BC. These locations represent wet-coastal and cold-dry climates, respectively. This simulation assumed 0.1% rain infiltration reaching the plywood surface and constant air cavity ventilation of 100 ACH. Figure 7a,b show the moisture content of plywood for the different types of exterior insulations of two-inch and four-inch thicknesses, respectively. In all four exterior insulation cases, the plywood moisture content is lower in Winnipeg cases than those in Vancouver. This is due to Vancouver’s higher annual rainfall. In both two-inch and four-inch thicknesses of PIR insulation with aluminum foil, the moisture content of the plywood exceeded 18%. As shown in Figure 7a, the maximum moisture content of the plywood in wall systems with two-inch fiberglass-faced PIR for Winnipeg and Vancouver was 17.78% and 19.71%, respectively. However, in the case of four-inch-thick fiberglass-faced PIR, the maximum moisture content of the plywood for Winnipeg and Vancouver cases was 16.70% and 18.47%, respectively, as shown in Figure 7b. The moisture content of the plywood in all wall systems with no-facer PIR insulation remained below 15% throughout the simulation period.

## 5. Conclusions

This study characterized the thermal and hygrothermal property of a PIR insulation with different facers. Based on the measured material property data, the long-term hygrothermal performance assessment of PIR insulation as rigid exterior insulation was examined. The experimental measurements show that most properties (such as density, water permeability and sorption isotherm) vary significantly as the thickness increases due to PIR being a composite of facer and PIR core. The material data presented here can be used in future modeling works to accurately simulate the hygrothermal property of PIR board with different thicknesses and facer materials.

In addition, this study examined specific application parameters such as thickness and facer types of the insulation core, simulations with varied rain infiltration rates, vapor barrier applications, and climates. Results show that thicker insulation provided a better moisture control strategy because it helped the sheathing board to stay warm. Facer materials used in the PIR insulation significantly affected the hygrothermal performance of wall systems. The hygrothermal performance of the PIR board PIR with unfaced insulation outperformed the XPS and the PIR Boards with aluminum and fiberglass facers. In light of its superior thermal performance in comparison to that of most foam insulation boards, PIR insulation with no facer or vapor-open facer material could contribute significantly to the current demands of high-performance, durable building construction practices.

## Figures and Tables

**Table 1:** Density measurement of Polyisocyanurate (PIR) board.

PIR Facer Type	Nominal Thickness mm (in.)	Actual Thickness mm (in.)	Density Kg/m^3^
Fiberglass facer	25.4 (1)	23.9 mm (15/16)	55.14 ± 0.42
	12.7 (0.5)	12.2 (0.48)	104.07 ± 6.33
Aluminum facer	38.1 (1.5)	36.7 mm (1–7/16)	34.3 ± 0.51
No facer core board	38.1 (1.5)	36.7 mm (1–7/16)	27.86 ± 0.48

**Table 2:** Thermal conductivity of PIR board.

Facer Type	Specimen Thickness mm	Mean Temperature °C	Hot Plate Temperature °C	Cold Plate Temperature °C	Thermal Conductivity W/(m·K)	Thermal Resistance m^2^·K/W
Fiberglass	24.12 ± 0.04	24.00 ± 0.03	34.01 ± 0.04	14.01 ± 0.04	0.026 ± 0.0007	0.944 ± 0.031
Aluminum facer	24.98 ± 0.02	24.02 ± 0.00	34.02 ± 0.00	14.02 ± 0.00	0.024 ± 0.0008	1.072 ± 0.038
No facer	24.86 ± 0.02	23.95 ± 0.05	33.96 ± 0.05	13.95 ± 0.05	0.023 ± 0.0008	1.076 ± 0.047

**Table 3:** Heat capacity of PIR board.

PIR Facer	Volumetric Heat Capacity J/(m^3^·K)	Specific Heat Capacity J/(kg·K)
Fiberglass facer	62943 ± 1083	1144 ± 23
Aluminum facer	49357 ± 1058	1439 ± 31
No facer	38090 ± 944	1257 ± 14

**Table 4:** Equilibrium moisture contents of PIR board samples.

RH, %	Moisture Content, kg/m^3^	
	Fiberglass Facer PIR	No Facer PIR
50	0.48 ± 0.02	0.36 ± 0.008
70	0.63 ± 0.03	0.49 ± 0.009
80	0.72 ± 0.02	0.55 ± 0.009
90	0.94 ± 0.01	0.68 ± 0.004
95	1.05 ± 0.02	0.77 ± 0.006
100	11.30 ± 0.262	8.79 ± 0.468

**Table 5:** Vapor permeability of PIR board.

Sample Mean RH (%)	Chamber RH (%)	Cup RH (%)	Permeability kg/(s·m·Pa)	
			PIR with Fiberglass Facer	No Facer PIR
25	50	0	9.813 × 10^−13^ ± 8.897 × 10^−13^	2.732 × 10^−12^ ± 1.789 × 10^−13^
35	70	0	1.110 × 10^−12^ ± 5.045 × 10^−13^	2.849 × 10^−12^ ± 1.711 × 10^−13^
45	90	0	1.28 × 10^−12^ ± 1.009 × 10^−13^	3.022 × 10^−12^ ± 1.786 × 10^−13^
75	50	100	1.570 × 10^−12^ ± 1.443 × 10^−13^	3.223 × 10^−12^ ± 1.287 × 10^−13^
85	70	100	2.408 × 10^−12^ ± 2.418 × 10^−13^	3.620 × 10^−12^ ± 1.375 × 10^−13^

**Table 6:** Simulation parameters.

	Parameter
**Climate**	Vancouver, BC (Zone 4C)
	Winnipeg, MB (Zone 7)
**Exterior insulation (type)**	PIR with aluminum foil
	PIR with fiberglass facing
	PIR with no facing
	XPS
**Exterior insulation (thickness)**	2 in.
	4 in.
**Rain infiltration**	0%
	0.1%
	0.5%

**Table 7:** Material properties used for simulation.

Material	Density (kg/m^3^)	Porosity (m^3^/m^3^)	Heat Capacity (J/kgK)	Thermal Conductivity (W/mK)	Diffusion Resistance Factor (-)
Stucco	1955.5	0.225	840	0.399	355.7
XPS	28.6	0.99	1470	0.025	170.56
Plywood	470	0.69	1880	0.084	1078.2
Spun bonded Polyolefin membrane	448	0.001	1500	2.4	328.4
Fiberglass	30	0.99	840	0.035	1.3

## References

[1] Ibrahim M., Biwole P.H., Wurtz E., Achard P. (2014). A study on the thermal performance of exterior walls covered with a recently patented silica-aerogel-based insulating coating. Build. Environ..

[2] Isaia F., Fantucci S., Capozzoli A., Perino M. (2015). Vacuum insulation panels: Thermal bridging effects and energy performance in real building applications. Energy Procedia.

[3] Kossecka E., Kosny J. Effect of insulation and mass distribution in exterior walls on dynamic thermal performance of whole buildings. Proceedings of the Thermal Performance of the Exterior Envelopes of Buildings VII.

[4] Khoukhi M. (2018). The combined effect of heat and moisture transfer dependent thermal conductivity of polystyrene insulation material: Impact on building energy performance. Energy Build..

[5] Kochkin V., Wiehagen J. (2017). Construction Guide to Next-Generation High-Performance Walls in Climate Zones 3-5-Part 1: 2x6 Walls (No. DOE/EE-1673-1).

[6] Li Y., Dang X., Xia C., Ma Y., Ogura D., Hokoi S. (2020). The effect of air leakage through the air cavities of building walls on mold growth risks. Energies.

[7] Ferdyn-Grygierek J., Kaczmarczyk J., Blaszczok M., Lubina P., Koper P., Bulińska A. (2020). Hygrothermal risk in museum buildings located in moderate climate. Energies.

[8] Ge H., Straube J., Wang L., Fox M.J. (2019). Field study of hygrothermal performance of highly insulated wood-frame walls under simulated air leakage. Build. Environ..

[9] Ibrahim M., Wurtz E., Biwole P.H., Achard P., Sallee H. (2014). Hygrothermal performance of exterior walls covered with aerogel-based insulating rendering. Energy Build..

[10] Fox M., Straube J., Ge H., Trainor T. Field test of hygrothermal performance of highly insulated wall assemblies. Proceedings of the 14th Canadian Conference on Building Science and Technology.

[11] Kočí V., Maděra J., Černý R. (2012). Exterior thermal insulation systems for AAC building envelopes: Computational analysis aimed at increasing service life. Energy Build..

[12] Langmans J., Klein R., Roels S. (2012). Hygrothermal risks of using exterior air barrier systems for highly insulated light weight walls: A laboratory investigation. Build. Environ..

[13] Pescari S., Tudor D., Tölgyi S., Măduţa C. (2015). Study Concerning the Thermal Insulation Panels with Double-Side Anti-Condensation Foil on the Exterior and Polyurethane Foam or Polyisocyanurate on the Interior. Key Eng. Mater..

[14] Wang L., Ge H. (2018). Stochastic modelling of hygrothermal performance of highly insulated wood framed walls. Build. Environ..

[15] (2013). Handbook, ASHRAE: Fundamentals 2013.

[16] Burch D.M., Desjarlais A.O. (1995). Water Vapor Measurements of Low Slope Roofing Materials.

[17] Kosmela P., Hejna A., Suchorzewski J., Piszczyk Ł., Haponiuk J.T. (2020). Study on the structure-property dependences of rigid pur-pir foams obtained from marine biomass-based biopolyol. Materials.

[18] Borowicz M., Paciorek-Sadowska J., Lubczak J., Czupryński B. (2019). Biodegradable, flame-retardant, and bio-based rigid polyurethane/polyisocyanurate foams for thermal insulation application. Polymers.

[19] Berardi U., Madzarevic J. (2020). Microstructural analysis and blowing agent concentration in aged polyurethane and polyisocyanurate foams. Appl. Therm. Eng..

[20] Mukhopadhyaya P., Bomberg M., Kumaran M., Drouin M., Lackey J., Van Reenen D., Normandin N. (2009). Long-term thermal resistance of polyisocyanurate foam insulation with impermeable facers. Insulation Materials: Testing and Applications: 4th Volume.

[21] Marrucho I.M., Santos F.M., Oliveira N., Dohrn R. (2005). Aging of Rigid Polyurethane Foams: Thermal Conductivity of N2 and Cyclopentane Gas Mixtures. J. Cell. Plast..

[22] Bogdan M., Hoerter J., Moore F.O. (2005). Meeting the insulation requirements of the building envelope with polyurethane and polyisocyanurate foam. J. Cell. Plast..

[23] Stovall T. (2012). Closed Cell Foam Insulation: A Review of Long Term Thermal Performance Research.

[24] Makaveckas T., Bliūdžius R., Burlingis A. (2020). The Influence of Different Facings of Polyisocyanurate Boards on Heat Transfer through the Wall Corners of Insulated Buildings. Energies.

[25] ASTM Standard C518-17 (2017). Standard Test Method for Steady-State Thermal Transmission Properties by Means of the Heat Flow Meter Apparatus.

[26] ASTM C1794-19 (2019). Standard Test Methods for Determination of the Water Absorption Coefficient by Partial Immersion.

[27] ASTM C1498-04a (2016). Standard Test Method for Hygroscopic Sorption Isotherms of Building Materials.

[28] ASTM E96/E96M-16 (2016). Standard Test Methods for Water Vapor Transmission of Materials.

